# Hearing Loss and Modifiable Dementia Risk Factors in Adults Aged 50 Years or Older Living in Tasmania, Australia

**DOI:** 10.1111/ajag.70204

**Published:** 2026-07-03

**Authors:** Mohammad Shoaib Hamrah, Kathleen Doherty, Aidan Bindoff, Lynette R. Goldberg, Jane Alty, Alex Kitsos, Eddy Roccati, Claire Eccleston, Anna King, Thomas Gregor Issac, Hiroshi Yatsuya, James Clement Vickers

**Affiliations:** ^1^ Wicking Dementia Research and Education Centre University of Tasmania Hobart Tasmania Australia; ^2^ Royal Hobart Hospital Hobart Tasmania Australia; ^3^ Tasmanian School of Medicine ‐ University of Tasmania Hobart Tasmania Australia; ^4^ Centre for Brain Research Indian Institute of Science Bangalore India; ^5^ Department of Public Health and Health System Nagoya University Graduate School of Medicine Nagoya Japan

**Keywords:** Australia, dementia, hearing loss, risk factors

## Abstract

**Objective:**

This study examined the associations between hearing loss (HL) and other modifiable dementia risk factors in adults aged 50 years or older living in Tasmania and explored the effects of corrected versus uncorrected HL on these factors.

**Methods:**

A cross‐sectional study design using an online survey categorised participants as No‐HL, HL‐corrected or HL‐uncorrected. Logistic regression estimated the odds of being in higher dementia risk categories.

**Results:**

The HL‐corrected group was more likely to be in the low‐risk alcohol group (OR = 0.567, 95% CI: 0.468–0.687), 28% less likely for high cholesterol (OR = 0.722, 95% CI: 0.571–0.913) and 26% less likely for diabetes (OR = 0.743, 95% CI: 0.582–0.948). They also had lower odds than the HL‐uncorrected group for alcohol risk (OR = 0.652, 95% CI: 0.508–0.837).

**Conclusions:**

Corrected HL is associated with reduced odds of higher‐risk categories for diabetes, alcohol use and high cholesterol.

## Policy Impact

The findings highlight the importance of integrating hearing rehabilitation into chronic disease care, promoting equitable access and supporting healthy ageing. They inform clinical practice, guide health policy to reduce service disparities and provide a foundation for future research exploring long‐term impacts of hearing rehabilitation on cognitive, cardiovascular and metabolic health.

## 1 Introduction

Adult‐onset hearing loss (HL) is the third‐most prevalent health condition among older adults, following heart disease and arthritis [1]. More than 42% of individuals with HL are aged 60 years or older, with prevalence increasing significantly with age [2]. In Australia, more than half of adults in their 60s experience HL, increasing to over 80% in those aged 80 years and above [3]. Given its high prevalence and broad impact, HL has been identified as a major contributor to disability among older adults living in Australia, [4] with an estimated annual economic cost of A$33.3 billion [5]. Hearing loss is also associated with reduced social engagement, impaired physical functioning and mental health challenges, [6] underscoring its significance as both a public health and ageing‐related concern.

Hearing loss is independently associated with several chronic conditions that are common in older adults, including dementia, diabetes, hypertension and hypercholesterolaemia [7, 8]. The aetiology of age‐related HL involves a complex interplay of genetic, environmental and lifestyle factors, including age‐related degeneration of the cochlear nerve, noise exposure and ototoxic medications [9]. Importantly, HL is a potentially modifiable risk factor for dementia [8] and therefore a target for interventions, such as hearing aids or cochlear implants, that may enhance auditory function and mitigate the risk of cognitive decline [10]. However, hearing rehabilitation remains underutilised in Australia [6].

Tasmania has the oldest population of any Australian state or territory, with a median age of 42.3 years [11] and a high prevalence of chronic diseases and dementia risk factors, including smoking (12%), obesity (71%) and hypertension (29%) [12]. Lower educational attainment and limited access to specialist services can reduce health literacy, limit awareness of preventive measures and hinder timely diagnosis and management of health conditions. These factors, which particularly occur in regional areas such as Tasmania, may further exacerbate the burden [13].

Hearing loss may contribute to cognitive decline through mechanisms such as reduced sensory input, social isolation and increased cognitive load [8]. Despite its importance, research examining HL in the context of overall dementia risk remains limited. Although HL is recognised as a contributor to cognitive decline, dementia and poor brain health in older adults, most studies have focussed on it in isolation or on a single risk factor [8]. This study addressed this gap and aimed to provide a novel perspective by investigating the association between HL (uncorrected HL) and the spectrum of modifiable dementia risk factors using the Dementia Risk Profile (DRP) to assess nine distinct risk domains [14]. Our approach considered multiple factors simultaneously, including physical and cognitive activity, diet, obesity, hypertension, dyslipidaemia, diabetes, smoking and alcohol use [14].

In addition, recent evidence indicates that HL is a consistent global risk factor for dementia [8] and interventions such as hearing aids have been shown to reduce cognitive decline in high‐risk older adults with poor hearing [10]. These findings suggest that if HL contributes to cognitive decline and interacts with other modifiable risk factors, individuals with uncorrected HL may bear a higher cumulative dementia risk. This underscores the importance of including both HL and its management in comprehensive risk assessments and dementia prevention strategies [15].

This study aimed to investigate the associations between HL and the prevalence and management of other modifiable dementia risk factors among Tasmanian adults 50 years and older, and explored the effects of corrected versus uncorrected HL on these factors. We hypothesised that individuals with uncorrected HL would report a greater number of modifiable dementia risk factors than those with No‐HL or corrected HL.

## 2 Methods

### 2.1 Participants and Data Collection

Participants were recruited from the Island Study Linking Ageing and Neurodegenerative Disease (ISLAND), a large‐scale, ongoing population‐based cohort study based in Tasmania, Australia. ISLAND aims to identify and mitigate modifiable dementia risk among adults 50 years and older [14]. Recruitment was conducted via print and social media campaigns, as well as community engagement initiatives. Eligibility criteria required participants to be residents of Tasmania, aged 50 years or older and to have access to the internet. Participants provided informed consent online and completed surveys between October 2019 and March 2025 (a period of five and a half years). Annual invitations to complete surveys were sent each October for the duration of the study.

The study was conducted according to the guidelines of the Declaration of Helsinki and approved by the University of Tasmania Health and Medical Human Research Ethics Committee (HREC H40267). Informed consent was obtained from all participants. Analyses used data from participants who completed the baseline survey and at least one full follow‐up survey, with the most recent in October 2025.

**FIGURE 1:**
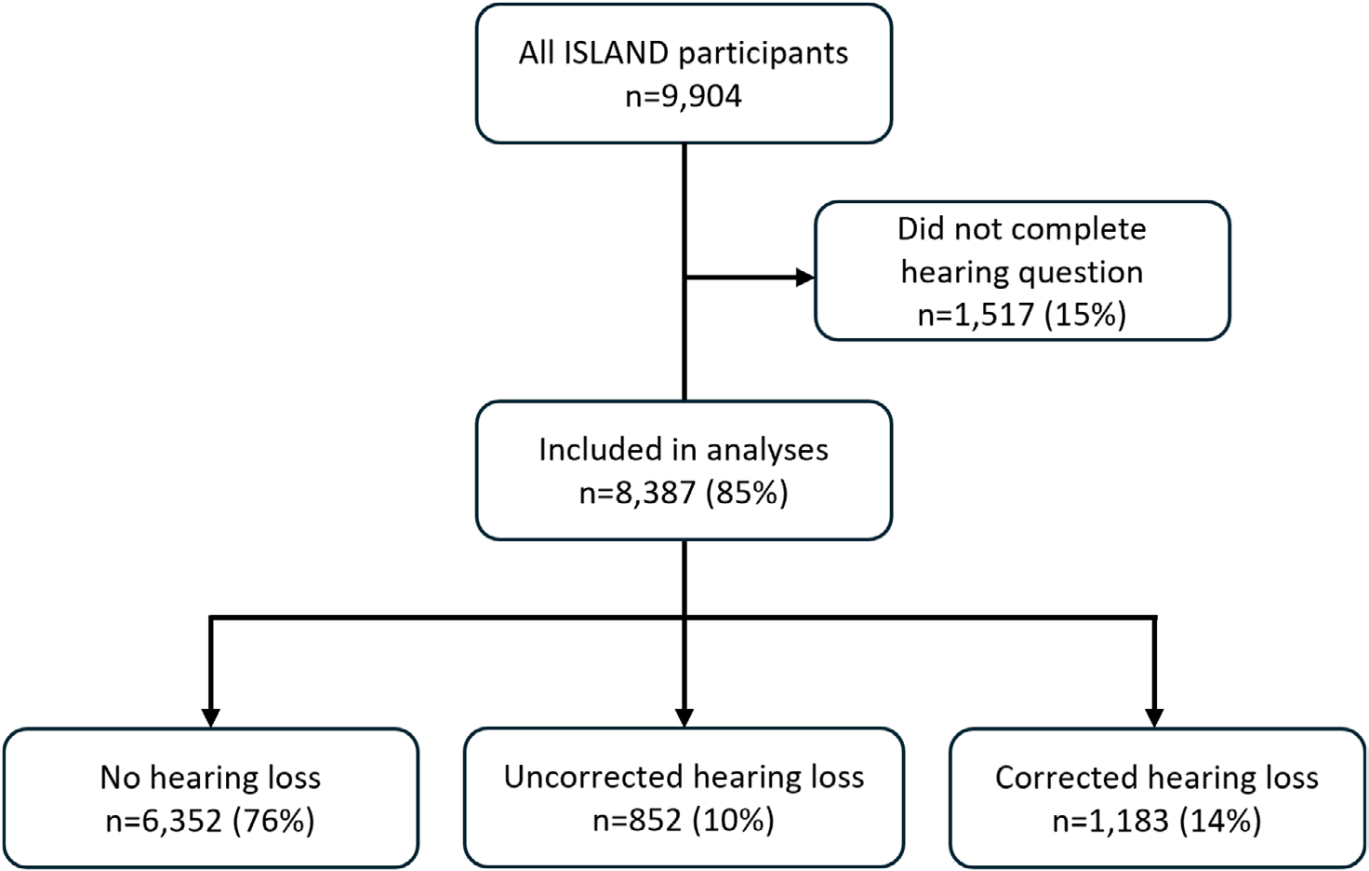
Overview of the Island Study Linking Ageing and Neurodegenerative Disease (ISLAND) study.

Variables collected comprised demographic characteristics, lifestyle factors and Dementia Risk Profile (DRP) scores. The DRP is a tool that provides a personalised assessment of dementia risk level (low, medium or high) across nine behavioural and lifestyle domains. These domains include physical and cognitive activity, diet, obesity, hypertension, dyslipidaemia, diabetes, smoking and alcohol consumption. The DRP helps participants track changes in their dementia‐related behaviours over time, encourages proactive management of risk and can be shared with their primary healthcare provider to support informed risk factor management [8, 14, 16, 17]. The DRP is based on the Lancet Commission on dementia prevention, intervention and care (2020), [18] and was developed before the latest update identifying 14 modifiable dementia risk factors [8]. The DRP has been shown to be sensitive to changes when people improve their health behaviours in an Australian population. Participant recruitment is illustrated in Figure 1.

## 3 Measures

### 3.1 Demographic Variables

Potential demographic variables evaluated in this study included age, gender and educational attainment (classified as university‐qualified, post‐secondary or school‐only).

### 3.2 Hearing Status

Hearing status was assessed via self‐report, which has demonstrated reasonable validity for population‐level screening compared to audiometric gold standard measures [19, 20]. The sample was divided into two groups based on their binary responses (***‘*** yes ***’*** or ***‘*** no ***’***) to the question, ***‘*** Do you have a hearing impairment? ***’*** Participants who reported hearing impairment were further classified based on their response to the question, ***‘*** Is your hearing impairment satisfactorily corrected, for example, with a hearing aid or other assistive listening device? ***’*** Hearing status was subsequently classified as a categorical variable with three levels: participants who reported no hearing loss (No‐HL), those with HL that was not corrected and those with HL that was corrected to their satisfaction (HL‐corrected), with No‐HL designated as the reference category.

### 3.3 DRP Data

Cognitive activity was assessed by the frequency of 11 different cognitive and cultural activities, with risk levels determined by total score: low risk = 33 or higher; high risk = less than 33. Physical activity was measured in minutes per week of light, moderate and vigorous activity, with each minute assigned 3.3, 4 or 8 metabolic equivalents of tasks (METs) respectively, and summed for a total score: low risk = 600 MET‐minutes or more; high risk = less than 600 MET‐minutes. Alcohol consumption was measured by the number of standard drinks per week: medium risk = 14 or fewer standard drinks per week combined with either more than two drinks per occasion or a report of binge drinking; high risk = more than 14 standard drinks per week.

Blood pressure management was determined based on hypertension diagnosis, check‐up frequency and management: low risk = no diagnosis with regular check‐ups, or a diagnosis that is medically managed; medium risk = unsure of diagnosis but with regular check‐ups, or diagnosis with regular check‐ups and partial/ongoing management; high risk = diagnosis without regular check‐ups and/or without medical management, or no diagnosis and no regular check‐ups.

Cholesterol management was assessed using the same criteria as blood pressure management, based on diagnosis, check‐up frequency and medical management: Risk levels were classified identically to blood pressure management (low, medium, high). Diabetes management was similarly evaluated based on diagnosis, check‐up frequency and medical management, with risk levels determined following the same approach as for blood pressure management. Body Mass Index (BMI) was calculated as weight in kilograms divided by height in metres squared, with risk categories defined as: low risk = 18–24.9; medium risk = 25–29.9 or less than 18; high risk = 30 or greater. Adherence to the Mediterranean‐DASH Intervention for Neurodegenerative Delay (MIND) diet was scored from 0 to 14: low risk = 12 or higher; medium risk = 7.5–11.9; high risk = less than 7.5. Smoking status was classified as: low risk = no smoking reported; medium risk = occasional smoking; high risk = daily, almost daily or weekly smoking [21].

### 3.4 Statistical Analysis

For statistical analysis, we used the baseline DRP results reported by each participant from completed surveys conducted between October 2019 and March 2025. Participants were divided into three categories depending on whether they had never reported HL from baseline to most recent survey (No‐HL), had reported HL but had never received correction for their HL and had reported both HLand HL‐correction when completing the survey (HL‐corrected). The DRP risk categories were dichotomised into ‘low’ and ‘medium/high’ (‘med/high’) for logistic regression analysis.

We used Firth's bias‐corrected, penalised logistic regression to estimate the log‐odds of med/high risk in each modifiable dementia risk domain for each category of HL, adjusted for age, gender and highest level of education. Firth's method was used to improve the numerical stability of estimates where separation might otherwise cause issues for unpenalised logistic regression. Omnibus test statistics were computed using penalised likelihood ratios. Where post hoc contrasts are presented, we used the Tukey method to control the family‐wise error rate (two‐sided statistical significance *p*_adj_ < 0.05). Estimates are reported with adjusted 95% confidence intervals (CI). Missing data were excluded from analyses. All statistical analyses were performed using the R environment for statistical computing [22].

## 4 Results

A total of 8387 participants were surveyed at baseline, with 852 in the HL group and 1183 in the HL‐corrected group (Table 1), corresponding to prevalence estimates of 10% and 14%, respectively.

**TABLE 1:** Demographic profile of the participants.

Characteristic	No HL (*n* = 6352)	HL (*n* = 852)	HL‐corrected (*n* = 1183)
Age, median (25th, 75th percentile)	62 (57, 68)	65 (60, 70)	71 (65, 75)
Gender			
Female	4796 (76)	484 (57)	678 (57)
Male	1546 (24)	362 (42)	501 (42)
Other/prefer not to say	10 (0)	6 (1)	4 (0)
Education			
University qualification	3285 (52)	422 (50)	554 (47)
Post‐secondary	1924 (30)	276 (32)	375 (32)
School only	935 (15)	127 (15)	212 (18)
Unknown	208 (3)	27 (3)	42 (4)
Year of participation			
(2019–2025), mean	6352 (14)	852 (14)	1183 (14)

*Note: n* (%).

Abbreviations: HL, Hearing loss; HL‐corrected, Hearing loss corrected; No HL, no hearing loss.

Participants in the HL and HL‐corrected groups were 32.9% and 33.1% more likely to be male, respectively (*p*_*adj*_ < 0.001). Participants in the HL‐corrected group were on average 7.1 years older than participants in the No‐HL group (95% CI: 5.0, 9.1) and 5.3 years older than participants in the HL group (95% CI: 2.3, 8.2) (*p*_*adj*_ < 0.001). There was also evidence of an association between education and HL (unadjusted *p* = 0.04). All of these variables were included as covariates in subsequent analyses. Three of the DRP modifiable dementia risk domains were significantly associated with HL: Alcohol (*p*_*adj*_ < 0.001), Cholesterol (*p*_*adj*_ = 0.009), and Diabetes (*p*_*adj*_ = 0.01). Results for all nine domains are presented in Table 2.

**TABLE 2:** Associations between self‐reported HL and modifiable risk factors.

Dementia risk factor	*χ*2 (df = 2)	HL‐corrected vs HL	HL‐corrected vs No‐HL	HL vs No‐HL
Alcohol risk	50	0.65 [0.51, 0 0.84]‐ < 0.001	0.57 [0.47, 0.69]‐ < 0.001	0.87 [0.72, 1.05]‐0.21
BMI risk	9.3	0.92 [0.57, 1.47]‐0.90	0.87 [0.61, 1.25]‐0.64	0.95 [0.66, 1.36]‐0.94
Cholesterol risk	19.6	0.79 [0.57, 1.09]‐0.19	0.72 [0.57, 0.91]‐0.003	0.91 [0.71, 1.17]‐0.67
Cognitive activity risk	18.2	0.92 [0.73, 1.17]‐0.71	1.02 [0.86, 1.21]‐0.97	1.10 [0.91, 1.34]‐0.46
Diabetes risk	11.5	0.75 [0.54, 1.03]‐0.09	0.75 [0.58, 0.95]‐0.01	1.00 [0.78, 1.29]‐ > 0.99
Hypertension risk	6.4	0.92 [0.57, 1.47]‐0.90	0.87 [0.60, 1.25]‐0.64	0.95 [0.66, 1.36]‐0.94
Mediterranean diet risk	5.9	0.94 [0.71, 1.23]‐0.84	1.02 [0.84, 1.23]‐0.98	1.09 [0.87, 1.35]‐0.66
Physical activity risk	0.2	1.01 [0.68, 1.52]‐ > 0.99	1.08 [0.81, 1.44]‐0.80	1.07 [0.76, 1.48]‐0.89
Smoking risk	19.8	1.31 [0.64, 2.67]‐0.65	0.87 [0.52, 1.44]‐0.79	0.66 [0.38, 1.14]‐0.18
		OR [95% CI]‐*p* value		

Abbreviations: BMI, body mass index; CI, confidence interval; df, degrees of freedom; HL, Hearing loss; HL‐corrected, Hearing loss corrected; No‐HL, no hearing loss; OR, Odds Ratio; *p*, *p*‐value; *χ* ^2^, chi‐square.

Participants in the HL‐corrected group were more likely to be in the lower‐risk alcohol consumption categories than those in the No‐HL group (OR = 0.567 [95% CI: 0.468, 0.687]) and the HL group (OR = 0.652 [95% CI: 0.508, 0.837]) after adjustment for age, gender and education. Participants in the HL‐corrected group also were more likely to be in the lower‐risk cholesterol category than those in the No‐HL group (OR = 0.722 [95% CI: 0.571, 0.913]). A similar pattern was observed compared with the HL group, although this difference was not statistically significant (OR = 0.791 [95% CI: 0.575, 1.087]).

Participants in the HL‐corrected group were more likely to be in the lower‐risk diabetes category than those in the No‐HL group (OR = 0.743 [95% CI: 0.582, 0.948]). A comparable trend was observed compared with the HL group, although this difference was not statistically significant (OR = 0.744 [95% CI: 0.536, 1.033]).

## 5 Discussion

This study investigated the associations between HL and other modifiable dementia risk factors among adults 50 years and older living in Tasmania and explored the effects of corrected versus uncorrected HL on these factors. We hypothesised that individuals with HL would have more modifiable dementia risk factors than those without HL or those with corrected HL. We found that participants with self‐reported HL had different profiles of dementia risk compared with participants with No‐HL, with lower risk in three of the nine domains—alcohol, cholesterol and diabetes. Participants in the HL‐corrected group tended to be older than the other groups and were more likely to be male than participants who did not report HL, although not more likely than participants who reported uncorrected HL. Our statistical analyses adjusted for age and gender, so any differences observed are unlikely to be due to confounding by these variables.

The present study revealed that individuals in the HL‐corrected group had reduced odds of being in the higher‐risk group for diabetes. This finding aligns with a prior cross‐sectional study using data from the third wave of the European Health Interview Survey (EHIS), which included 17,660 participants aged 15 years and older with hearing impairment across 28 European countries, showing that hearing‐aid users had a lower risk of diabetes compared with non‐users [23]. Several factors might help explain this association. For instance, hearing‐aid use could support social engagement and reduce communication barriers, which may promote better self‐care, adherence to medical recommendations and engagement in health‐promoting behaviours such as diet, exercise and medication management [24, 25]. Higher social engagement is associated with higher socioeconomic status, (SES) as individuals with higher SES generally have greater access to material resources, social networks and communication opportunities that facilitate participation in social activities [26]. In contrast, lower SES is also associated with lower rates of hearing aid use and reduced access to hearing services, which may further limit opportunities for social engagement [26, 27]. However, the analysis did not adjust for participants' SES, which may influence both hearing‐aid uptake and health outcomes. These findings underscore the importance of considering hearing‐aid interventions not only for auditory rehabilitation but also as a potential strategy to promote broader health benefits, including improved metabolic and chronic disease management.

Our analysis demonstrated that individuals in the HL‐corrected group had reduced odds of being in the higher‐risk category for alcohol consumption, consistent with previous cross‐sectional research suggesting that HL rehabilitation is associated with lower alcohol use [23]. It is hypothesised that hearing aids may enhance communication and social engagement, which could reduce loneliness and social isolation, both of which are recognised risk factors for heavy drinking [24, 28].

The study found that individuals in the HL‐corrected group had reduced odds of being in the higher‐risk category for the cholesterol domain than participants in the No‐HL group, aligning with prior research linking metabolic syndrome—including high triglycerides and low high‐density lipoprotein (HDL) cholesterol—to an increased risk of hearing loss. Effective management of these conditions may help reduce this risk [29]. It is plausible that hearing‐aid use might promote healthier behaviours, such as increased physical activity and social engagement, which could hypothetically support lipid metabolism, raise HDL cholesterol and improve adherence to diet and medical care [30]. We did adjust for education, which is correlated with both SES and health literacy, but residual confounding from broader social factors cannot be ruled out. These potential pathways provide a speculative explanation for the observed association between hearing correction and reduced odds of hypercholesterolaemia. While these associations are not causal, the findings suggest the possibility that hearing rehabilitation may contribute to metabolic health in addition to auditory function, [31] highlighting a hypothesis that warrants further longitudinal and interventional investigation.

These findings highlight the importance of integrating hearing rehabilitation into chronic disease care and healthy ageing strategies. They emphasise equitable access, interdisciplinary collaboration and health literacy programs, particularly in regional areas. Future research should examine long‐term effects on metabolic, cardiovascular, cognitive and auditory outcomes, linking hearing management with modifiable dementia risk.

### 5.1 Limitations and Strengths

To begin with, the study's strengths include a population‐based sample, adjustment for potential confounding variables (age, gender and education), and the inclusion of regional and rural participants. Studying these populations is particularly important, as individuals in larger urban areas—with greater resources, a larger pool of social contacts and better access to support and healthcare—may experience different outcomes. In addition, a key strength of the study is the large sample size and the inclusion of multiple dementia risk domains. However, there is a potential for selection bias, as more health‐conscious individuals may be more likely to seek help for HL and engage in healthier behaviours. However, interpretation of findings from the ISLAND cohort may be challenging, as this cohort appears to be relatively healthier and potentially more financially advantaged than the general Tasmanian population [32]. Moreover, the study describes the role of HL and its influence on other cardiovascular risk factors within the DRP, highlighting broader implications for targeted treatment and policy development for these non‐communicable diseases, in addition to dementia.

However, several limitations should also be acknowledged. First, the cross‐sectional design precludes conclusions about causality between modifiable dementia risk factors and HL, highlighting the need for longitudinal studies to clarify the direction of these associations. Furthermore, both HL and modifiable dementia risk factors were assessed via self‐report rather than objective measures, which may have led to an underestimation of the true strength of these associations and introduced the possibility of response bias and potentially underestimated the strength of the observed associations. Additionally, there is a potential for selection bias. Individuals who seek help for HL and adopt hearing‐related interventions may also be more proactive in managing their overall health, including engagement in healthier lifestyle behaviours and preventive healthcare practices. Such individuals may therefore have a lower baseline risk of dementia independent of hearing‐related factors. Although our analyses adjusted for multiple demographic, clinical and lifestyle variables across several dementia risk domains, residual confounding and self‐selection bias may still have influenced the observed associations. Consequently, the findings should be interpreted with caution, and future longitudinal and interventional studies are needed to further clarify the causal relationship between hearing‐related healthcare engagement and dementia risk.

Another limitation is that information on duration and laterality of HL, which were not captured, as well as the potential effects of correction or treatment, have not been explored in detail. Moreover, information on the onset and correction of HL, as well as the type of correction used, was unavailable. In addition, the DRP used in this study was based on an earlier Lancet Commission list of risk factors [18] and does not fully align with the updated 14 risk factors of the latest Lancet Commission review [8]. The ISLAND cohort also exhibits potential biases, including a predominance of participants who are White, of Northern European ancestry and digitally literate, which may limit the generalisability of these findings. Additionally, unmeasured factors such as genetic predisposition, family history, occupational exposures and the absence of objective auditory acuity measures could have influenced the observed associations.

A further limitation is that detailed information regarding participants' hearing aid use was not systematically collected. Specifically, data on whether participants used unilateral or bilateral hearing aids, the specific type and technology of hearing aids, duration of hearing aid use, average daily usage hours and whether fittings were verified using real‐ear measurements were not available. These factors may influence hearing aid outcomes and participant experiences and therefore could have affected the findings of the study. In addition, the study did not include a standardised measure such as the International Outcome Inventory for Hearing Aids (IOI‐HA) to assess the effectiveness and benefit of hearing aid treatment from the participants' perspective. Future studies should incorporate these clinical and patient‐reported outcome measures to provide a more comprehensive evaluation of hearing aid use and its impact.

Moreover, hearing status in our study was based on self‐reported HL rather than objective audiometric thresholds. As a result, we were unable to classify the type and degree of HL with precision. Future research could use an instrument such as the IOI‐HA to assess hearing aid effectiveness. An additional limitation relates to item‐level missingness among participants who completed at least one DRP item. Overall, 7% of DRP items were not attempted, with comparable rates across groups: No‐HL (6.5%), HL (6.9%) and HL‐corrected (8%). This represents a relatively low and fairly balanced level of missingness, suggesting it is unlikely to have introduced systematic bias at the overall scale level. However, of the participants who completed at least one item on the DRP, 8% of HL‐corrected did not complete the Alcohol section compared to 4% HL and 5% No‐HL. To a similar but smaller degree, HL‐corrected had greater missingness for Cog Activity (5%) compared to 3% and 2% missing for HL and No‐HL, respectively. We have acknowledged this as a potential limitation, as differential missingness in these domains could introduce a small degree of bias for those specific items. A final limitation of the study is that missing data were not uniform across health literacy groups, particularly for the alcohol and cognitive activity domains. This uneven pattern may have introduced bias and should be considered when interpreting the findings. Further research is required to elucidate the underlying pathophysiological mechanisms linking HL to modifiable dementia risk factors, and to explore potential pharmacological or behavioural interventions targeting these mechanisms to prevent or reduce HL.

## 6 Conclusions

This large population‐based study provides important evidence on the associations between HL and several modifiable dementia risk factors among adults 50 years and older living in Tasmania. Individuals in the HL‐corrected group exhibited lower odds of being in the higher‐risk categories for diabetes, alcohol consumption and the cholesterol domain, suggesting that hearing rehabilitation may be associated with reduced risk for these factors. These findings highlight the value of integrating hearing rehabilitation into broader public health and chronic disease frameworks.

## Funding

This project was supported by funding from the Australian Medical Research Future Fund, St Luke's Health and the Tasmanian Masonic Centenary Medical Research Foundation.

## Ethics Statement

The study was conducted according to the guidelines of the Declaration of Helsinki and approved by the University of Tasmania Health and Medical Human Research Ethics Committee (H40267).

## Consent

Informed consent was obtained from all participants.

## Conflicts of Interest

The authors declare no conflicts of interest.

## Data Availability

The data that support the findings of this study are available on request from the corresponding author. The data are not publicly available due to privacy or ethical restrictions.
